# A de-identified inpatient clinical pathway dataset integrating ICD-10 diagnoses, ICD-9-CM procedures, Indonesian Case-Based Groups (INA-CBG), and length of stay for healthcare analytics

**DOI:** 10.12688/f1000research.187759.1

**Published:** 2026-10-14

**Authors:** Suryanto Nugroho, R.V. Hari Ginardi, I Ketut Eddy Purnama

**Affiliations:** 1 Doctoral Program in Technology Management,Interdisciplinary School of Management and Technology, Institut Teknologi Sepuluh Nopember, Surabaya, East Java, 60111, Indonesia; 2 Department of Informatics, Universitas Muhammadiyah PKU Surakarta, Surakarta, Jawa Tengah, 57137, Indonesia; 3 Department of Information Technology, Institut Teknologi Sepuluh Nopember, Surabaya, East Java, 60111, Indonesia; 4 Department of Computer Engineering, Institut Teknologi Sepuluh Nopember, Surabaya, East Java, 60111, Indonesia

**Keywords:** Information management, clinical pathway, ICD-10, ICD-9-CM, INA-CBG, length of stay, healthcare analytics, machine learning

## Abstract

**Background:**

Healthcare administrative databases have become indispensable resources for healthcare analytics, hospital management, clinical pathway analysis, health services research and artificial intelligence. Modern hospital information systems routinely collect standardized diagnoses, procedures, reimbursement classifications, and hospitalization outcomes in everyday clinical practice. Despite their importance, publicly accessible datasets integrating diagnosis codes, procedure codes, case-mix classifications, and hospitalization outcomes remain scarce, particularly in low- and middle-income countries (LMICs). The absence of comprehensive administrative datasets limits reproducible research in clinical pathway modelling, healthcare operations, reimbursement analysis, and machine learning. To address this gap, we developed and publicly released a de-identified inpatient clinical pathway dataset derived from routine hospital administrative records processed through the Indonesian Case-Based Groups (INA-CBG) reimbursement system.

**Methods:**

The dataset was constructed from finalized inpatient hospitalization records collected between November 2016 and April 2026. Clinical diagnoses were represented using the International Classification of Diseases, Tenth Revision (ICD-10), and clinical procedures were encoded using the International Classification of Diseases, Ninth Revision, Clinical Modification (ICD-9-CM). Each hospitalization episode included one primary diagnosis, up to 11 secondary diagnoses, one primary procedure, up to 16 secondary procedures, one INA-CBG classification code, one INA-CBG description, and one length-of-stay (LOS) variable. Data extraction, transformation, harmonization, de-identification, quality assessment, and validation were performed using a reproducible Python-based computational workflow. The final repository contained 165,391 hospitalization episodes represented by 32 standardized variables.

**Conclusions:**

The dataset provides an openly available resource for healthcare analytics, clinical pathway research, process mining, hospital management, reimbursement analysis, predictive modelling, explainable artificial intelligence, and healthcare operational research. The combination of standardized ICD-10 diagnoses, ICD-9-CM procedures, INA-CBG classifications, and hospitalization outcomes enables diverse secondary analyses while maintaining interoperability with existing healthcare information systems. All datasets, metadata, source codes, and supporting documentation are publicly available through Zenodo to facilitate transparency, reproducibility, and future methodological development.

## 1. Introduction

The digital transformation of healthcare has substantially increased the availability of routinely collected administrative and clinical data within hospital information systems, ^1^ ^,^ ^2^ Every inpatient admission generates structured electronic records describing the diagnoses, procedures, reimbursement information, and clinical outcomes. ^3^ These routinely collected data have become an important source of evidence for healthcare analytics, hospital performance evaluation, resource planning, clinical pathway optimization, and the development of artificial intelligence (AI) applications. ^4^ Compared with manually collected research datasets, administrative healthcare databases provide larger sample sizes, longer observation periods, and standardized coding systems that facilitate reproducible analyses across multiple healthcare domains. ^5^

Standardized clinical coding systems are fundamental components of hospital administrative databases because they ensure interoperability between clinical documentation, reimbursement mechanisms, and secondary research applications, ^6^ ^,^ ^7^ Diagnostic information is commonly represented using the International Classification of Diseases, Tenth Revision (ICD-10), whereas procedural interventions are encoded using the International Classification of Diseases, Ninth Revision, Clinical Modification (ICD-9-CM). ^8^ These internationally recognized coding systems provide consistent representations of diseases and procedures and support healthcare reimbursement, epidemiological surveillance, hospital benchmarking, and clinical research. ^9^

In Indonesia, hospital reimbursement for patients covered by the National Health Insurance (Jaminan Kesehatan Nasional, JKN) is performed using the Indonesian Case-Based Groups (INA-CBG) system. ^10^ The INA-CBG is a case-mix classification framework that assigns each hospitalization episode to a reimbursement category according to the diagnosis, procedures, severity level, and expected healthcare resource utilization. ^11^ Consequently, routine administrative records generated through the INA-CBG reimbursement workflow contain a rich combination of standardized diagnosis codes, procedure codes, case-mix classifications, and hospitalization outcomes. ^12^ These records provide valuable opportunities for secondary analyses involving healthcare operations, patient flow modelling, reimbursement evaluation, hospital efficiency, and predictive modelling. ^13^

During the past decade, numerous publicly available healthcare datasets have been released to support artificial intelligence and health informatics research. ^14^ However, most existing repositories focus on intensive care monitoring, physiological measurements, medical imaging, laboratory examinations, and disease-specific registries, ^15^ ^,^ ^16^ Public datasets that integrate standardized administrative coding systems, reimbursement classifications, and hospitalization outcomes remain relatively uncommon. ^17^ This limitation is particularly evident for datasets originating from low- and middle-income countries, where publicly available administrative healthcare repositories remain scarce despite the widespread implementation of electronic hospital information systems. ^18^

Administrative datasets constructed using standardized clinical coding systems offer several important advantages for healthcare research. ^5^ First, they facilitate investigations into diagnosis–procedure relationships and clinical pathway patterns across large patient populations. ^19^ Second, they enable the development and evaluation of predictive models for hospital outcomes, such as length of stay, resource utilization, and reimbursement categories. ^20^ Third, they support healthcare operations research, including bed management, discharge planning, capacity forecasting and process mining. ^21^ Finally, standardized coding systems improve interoperability and reproducibility by enabling researchers to compare findings across institutions using internationally recognized classifications. ^22^

To address the shortage of openly accessible administrative healthcare datasets, we developed a de-identified inpatient clinical pathway repository derived from routine hospital administrative records processed through the Indonesian INA-CBGs reimbursement system. The repository contains 165,391 inpatient hospitalization episodes collected over approximately ten years and organized into a standardized analytical structure comprising 32 variables, including ICD-10 diagnosis codes, ICD-9-CM procedure codes, INA-CBG classifications, INA-CBG descriptions, and length-of-stay information.

Unlike disease-specific datasets or electronic medical record repositories that primarily emphasize clinical measurements, the present repository focuses on standardized administrative information routinely generated during hospital reimbursement and clinical documentation. The dataset was intentionally designed to support a broad range of secondary applications, including healthcare analytics, machine learning, process mining, clinical pathway modelling, explainable artificial intelligence, reimbursement research, and hospital management. ^23^ By transforming variable-length diagnosis and procedure records into a harmonized fixed-column structure, the repository enables direct application in statistical software and machine-learning frameworks without extensive preprocessing.

In addition to the dataset itself, the repository includes comprehensive metadata, documentation, descriptive statistics, visualization outputs, and reproducible Python scripts that describe every stage of dataset construction. These supplementary materials promote transparency, facilitate independent verification, and encourage reuse by researchers, healthcare professionals, policy makers, and software developers. The objective of this Data Note is to describe the methodology used to construct the dataset, document its structure and validation procedures, and provide sufficient information to enable researchers to reuse the repository in future healthcare analytics and health informatics studies.

## 2. Materials and methods

### 2.1 Study design

Figure 1. Overview of the Dataset Construction Workflow.

**Figure 1.**
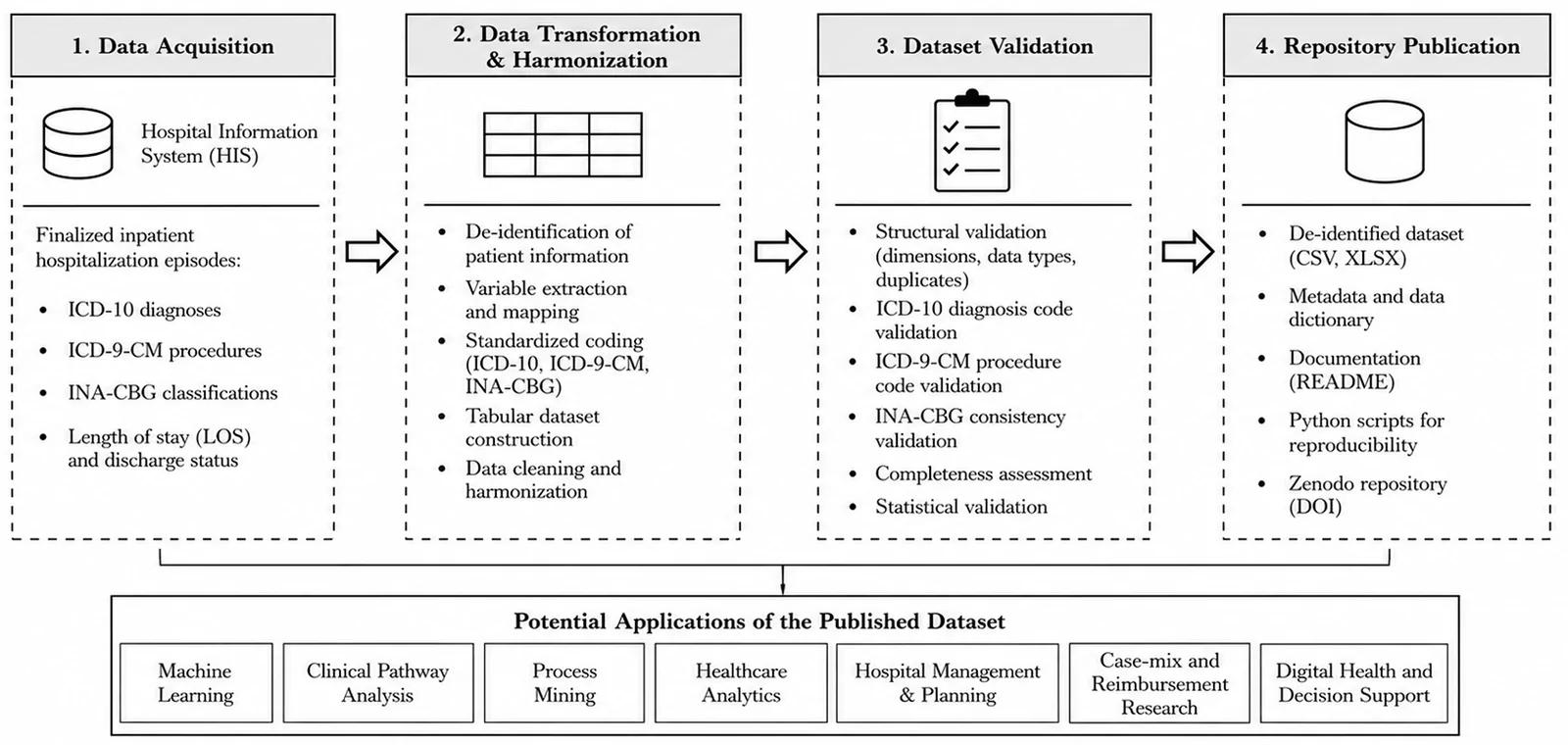
Overview of dataset construction workflow. The workflow illustrates the four-stage process used to construct a published repository. Finalized inpatient administrative records were acquired from the Hospital Information System (HIS), transformed into a standardized analytical dataset through de-identification and data harmonization, validated using structural and clinical coding quality assurance procedures, and finally published as an open-access repository with metadata, documentation, and reproducible Python scripts. Abbreviations: HIS, Hospital Information System; ICD-10, International Classification of Diseases, Tenth Revision; ICD-9-CM, International Classification of Diseases, Ninth Revision, Clinical Modification; INA-CBG, Indonesian Case-Based Groups; LOS, Length of Stay.

This study describes the development of a de-identified inpatient clinical pathway dataset derived from routinely collected hospital administrative data. The dataset was designed as a reusable research resource to facilitate healthcare analytics, clinical pathway analysis, machine learning, process mining, hospital operations research, and decision-support system development. Repository construction followed a reproducible data engineering workflow consisting of four sequential stages, as shown in Figure 1: (1) data acquisition, (2) data transformation and harmonization, (3) dataset validation, and (6) repository publication.

Unlike conventional observational clinical studies, this study did not aim to evaluate treatment effectiveness or investigate patient outcomes. Instead, the primary objective was to construct a standardized analytical dataset that accurately represents finalized inpatient administrative records while preserving the relationships among diagnoses, procedures, case-mix classifications, and hospitalization outcomes. ^24^

The overall workflow was designed to satisfy four major requirements: (i) preservation of standardized clinical coding, (ii) structural consistency across all hospitalization episodes, (iii) compatibility with statistical and machine-learning software, and (iv) reproducibility through publicly available source code and documentation.

### 2.2. Data source

The repository was constructed using routinely collected inpatient hospitalization records extracted from the Hospital Information System (HIS) of a tertiary referral hospital in Indonesia. The hospital implements a fully integrated electronic administrative workflow in which inpatient clinical documentation, diagnosis coding, procedure coding, reimbursement processing, and discharge information are managed digitally. ^25^

The original operational database contains comprehensive administrative information generated throughout the hospitalization process. ^26^ For this repository, only finalized inpatient episodes were included after the completion of the national reimbursement process. Restricting data extraction to finalized records ensured consistency in diagnoses, procedures, reimbursement classifications, and hospitalization outcomes.

Hospitalization episodes included in the repository were recorded between November 2016 and April 2026, representing approximately ten years of routine inpatient services across multiple medical specialties. During this period, the hospital consistently applied the Indonesian INA-CBG reimbursement system, allowing for a standardized representation of diagnoses, procedures, severity levels, and expected healthcare resource utilization. The source records consisted exclusively of de-identified administrative variables relevant to hospital reimbursement and health care analytics. Direct patient identifiers were excluded before repository construction to protect patient confidentiality while preserving the analytical value of clinical coding information.

### 2.3. Hospital administrative workflow

Understanding the administrative workflow underlying the dataset is essential because every variable contained in the repository originates from a standardized reimbursement process, rather than manual research data collection. The administrative workflow underlying the construction of the repository is illustrated in Figure 2.

**Figure 2.**
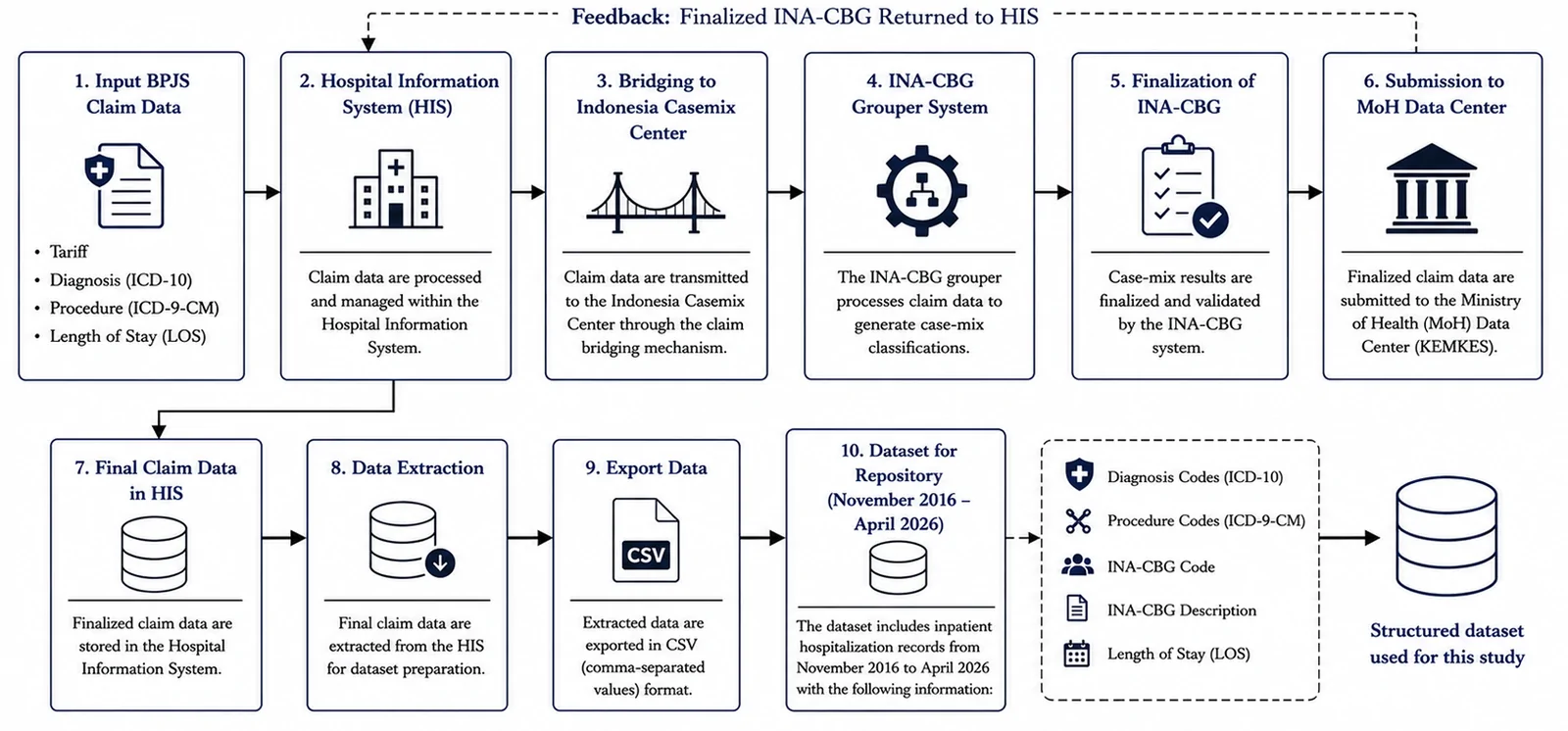
Hospital administrative workflow and data acquisition processes. The figure illustrates the routine inpatient administrative workflow beginning with patient admission, clinical documentation, diagnosis and procedure coding, INA-CBG reimbursement processing, claim verification, and extraction of finalized administrative records used for repository construction. Only finalized inpatient episodes were included to ensure consistency between the diagnoses, procedures, reimbursement classifications, and hospitalization outcomes. Abbreviations: HIS, Hospital Information System; EMR, Electronic Medical Record; ICD-10, International Classification of Diseases, Tenth Revision; ICD-9-CM, International Classification of Diseases, Ninth Revision, Clinical Modification; INA-CBG, Indonesian Case-Based Groups.

Figure 2. Overview of the Indonesian inpatient administrative workflow used for the repository construction.

Patients admitted to the hospital undergo routine clinical assessment, diagnostic investigations, therapeutic interventions, and continuous inpatient management. Throughout hospitalization, the attending physicians documented diagnoses and performed procedures within the electronic medical record. Upon discharge, certified clinical coders review the complete medical records and assign standardized ICD-10 diagnosis codes and ICD-9-CM procedure codes according to the national coding guidelines.

Following the completion of coding, administrative claim records are prepared for submission to the Indonesian National Health Insurance reimbursement system. Claims are processed using the Indonesian Case-Based Groups (INA-CBG) grouper, which assigns each hospitalization episode to a standardized reimbursement category based on diagnosis, procedures, disease severity, complications, comorbidities, and expected healthcare resource utilization.

After verification and reimbursement approval, the finalized claim records are returned to the Hospital Information System and simultaneously submitted to the Ministry of Health as part of the national reporting process. Only these finalized administrative records were used during repository construction, ensuring that every hospitalization episode contained validated diagnosis codes, procedure codes, reimbursement classifications, and hospitalization outcomes. This workflow minimizes the inconsistencies commonly observed in preliminary clinical documentation and provides a reliable foundation for secondary healthcare research.

### 2.4. Inclusion and exclusion criteria

Hospitalization episodes were selected according to predefined eligibility criteria to ensure the structural consistency and completeness of the published repository.

Inclusion criteria

Hospitalization records were included when the following conditions were satisfied:

1. Inpatient admissions were completed through the routine hospital discharge process.
2. finalized ICD-10 diagnosis coding was available.
3. finalized ICD-9-CM procedure coding, when applicable.
4. finalized INA-CBG reimbursement classification was assigned.
5. documented the length of stay in the hospital.
6. completed the administrative reimbursement workflow.

Exclusion criteria

1. Hospitalization records were excluded if they met any of the following criteria:
2. incomplete reimbursement processing.
3. duplicate hospitalization episodes.
4. administrative testing records.
5. corrupted or unreadable electronic record.
6. inconsistent reimbursement information identified during the quality assessment.

The application of these eligibility criteria ensured that the published repository represented validated inpatient hospitalization episodes suitable for secondary analysis.

### 2.5 Data extraction

Following the identification of eligible hospitalization episodes, administrative records were extracted directly from the Hospital Information System using structured database queries.

The extracted variables included the following:

1. primary diagnosis,
2. secondary diagnoses,
3. primary procedure,
4. secondary procedures,
5. INA-CBG code,
6. INA-CBG description,
7. hospitalization length of stay.

To minimize information loss, no aggregation or recoding of diagnostic and procedural variables was performed during extraction. Instead, the original standardized clinical codes assigned during hospital reimbursement were retained throughout the construction of the repository. Data extraction generated an initial administrative dataset before subsequent de-identification and transformation procedures.

### 2.6 Data de-identification

The protection of patient privacy is a fundamental requirement for the construction of a repository.

Prior to transformation, all direct patient identifiers were permanently removed from the extracted administrative data. The removed identifiers included:

1. patient name,
2. medical record number,
3. national identity number,
4. address,
5. telephone number,
6. insurance identification number,
7. date of birth,
8. physician identifiers,
9. other potentially identifiable administrative information.

No attempt was made to generate pseudonymous identifiers because longitudinal patient tracking was not required for the intended analytical application. Each row within the final repository, therefore, represents an anonymous hospitalization episode that cannot be linked back to an individual patient. The resulting repository complies with institutional data-sharing requirements for secondary research while substantially reducing the risk of disclosure.

### 2.7 Clinical coding systems

The repository integrates three complementary coding systems routinely used in Indonesian hospital administrative practices.

ICD-10 Diagnosis Coding

Diagnostic information was represented using the International Classification of Diseases, Tenth Revision (ICD-10). Each hospitalization episode contains one primary diagnosis describing the principal reason for admission, together with up to 11 secondary diagnoses documenting comorbidities, complications, and additional clinical conditions recorded during hospitalization. ^27^

ICD-9-CM Procedure Coding

Procedural information was represented using the International Classification of Diseases, Ninth Revision, Clinical Modification (ICD-9-CM). ^28^ One primary procedure was recorded for each hospitalization episode when applicable, followed by up to 16 secondary procedures documenting additional diagnostic or therapeutic interventions.

INA-CBG Classification

Case-mix information was represented using the finalized Indonesian Case-Based Group (INA-CBG) classifications generated by the national reimbursement system. Each hospitalization episode contains both the structured INA-CBG code and its corresponding textual description, allowing analyses based on either coded classifications or clinically interpretable categories. ^29^

The integration of these coding systems provides a comprehensive representation of inpatient administrative records while maintaining compatibility with the national reimbursement standards and international clinical coding practices.

### 2.8 Dataset structure

The published repository was designed to provide a standardized and machine-readable representation of routine inpatient administrative records while preserving hierarchical relationships among diagnoses, procedures, reimbursement classifications, and hospitalization outcomes. Rather than storing clinical information in a variable-length format, the dataset was organized into a fixed-column tabular structure to facilitate interoperability with statistical software, relational databases and machine learning frameworks.

Each row in the dataset represents a single completed inpatient hospitalization. Consequently, one hospitalization episode corresponded to one observation, ensuring that no admission was fragmented across multiple records. This episode-based organization allows researchers to directly analyze patient encounters without requiring additional data aggregation or restructuring.

The dataset contains 165,391 hospitalization episodes described by 32 standardized variables organized into four logical components: diagnosis, procedure, administrative, and hospitalization outcome variables. This organization reflects the natural sequence of clinical documentation and reimbursement processing within the hospital information system while maintaining compatibility with downstream analytical applications. ^30^

Diagnosis information is represented using 12 variables consisting of one Primary_Diagnosis field and 11 Secondary_Diagnosis fields encoded according to the International Classification of Diseases, Tenth Revision (ICD-10). The primary diagnosis represents the principal condition responsible for hospitalization, whereas secondary diagnosis variables record additional diseases, complications, or comorbidities that are documented during the same admission. Maintaining separate diagnosis fields preserves the hierarchical order assigned during routine clinical coding and avoids information loss that may occur when multiple diagnoses are concatenated into a single variable. ^31^

The procedural information follows an equivalent organizational strategy. One Primary_Procedure variable records the principal therapeutic or diagnostic intervention performed during hospitalization, while 16 Secondary_Procedure variables capture additional procedures encoded using the International Classification of Diseases, Ninth Revision, Clinical Modification (ICD-9-CM). By preserving the original procedural sequence, the dataset supports investigations into procedural complexity, treatment pathways, and healthcare resource utilization. ^32^

Administrative information generated through the Indonesian Case-Based Groups (INA-CBG) reimbursement system was represented using two dedicated variables. The first variable contains the finalized INA-CBG code assigned during the reimbursement processing, whereas the second provides the corresponding textual description of the case-mix category. These variables enable analyses based on either standardized reimbursement codes or clinically interpretable disease group descriptions. ^33^

Hospitalization outcomes were represented using a single Length_of_Stay (LOS) variable, expressed as the total duration of inpatient care in days. LOS was retained as an integer variable without transformation or categorization to preserve its original administrative meaning and maximize flexibility for subsequent statistical analyses and predictive modelling. ^34^

Because the number of diagnoses and procedures differs considerably among patients, the transformation workflow converts variable-length clinical records into a harmonized fixed-column representation. Hospitalization episodes containing fewer diagnoses or procedures retain empty values in the remaining secondary diagnosis or procedure field. These empty fields should not be interpreted as missing data; instead, they accurately indicate that no additional diagnoses or procedures were documented during the corresponding hospitalization episode.

The resulting dataset provides a consistent analytical structure that can be directly imported into commonly used software environments without requiring additional pre-processing. This standardized organization substantially improves reproducibility and enables diverse secondary applications, including healthcare analytics, clinical pathway analysis, process mining, machine learning, reimbursement studies, and hospital operations research. The organization of the 32 variables across the four dataset components is presented in Table 1.

**Table 1.** Organization of the published dataset.

Dataset Component	Variables	Description
Diagnosis	12	One primary diagnosis and eleven secondary diagnoses encoded using ICD-10
Procedure	17	One primary procedure and sixteen secondary procedures encoded using ICD-9-CM
Administrative	2	Final INA-CBG code and corresponding case-mix description
Outcome	1	Length of stay (LOS) recorded in days
Total	32	Standardized variables describing each hospitalization episode

Figure 3 illustrates the logical organization of the published datasets. The repository is structured hierarchically, beginning with diagnostic variables, followed by procedural variables, administrative case-mix information, and hospitalization outcomes. This organization preserves the original administrative workflow while providing a standardized tabular representation suitable for statistical analysis, healthcare analytics, and machine learning applications.

**Figure 3.**
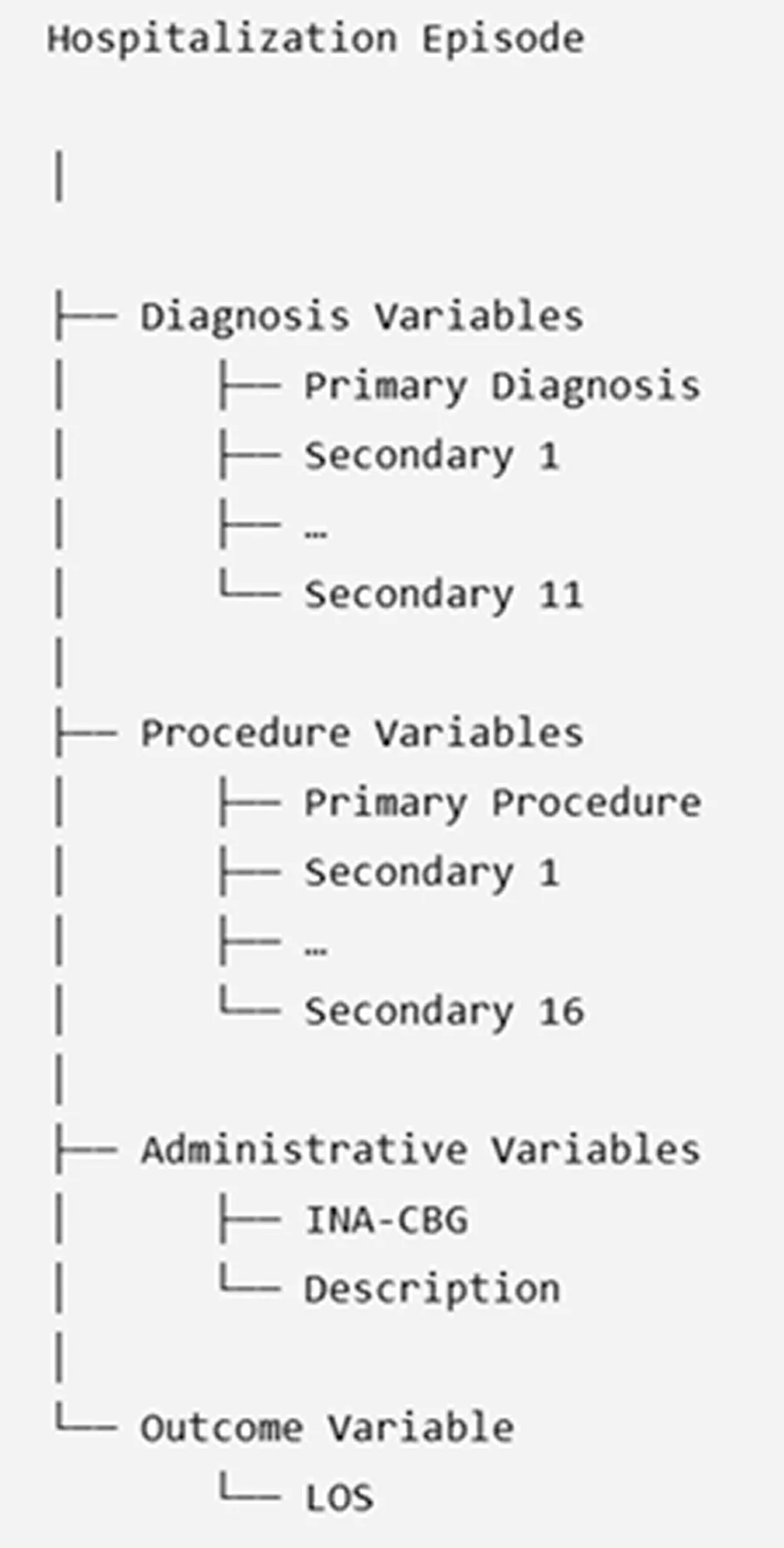
Organization of the published inpatient clinical pathway dataset. The figure presents the logical organization of the dataset into four principal components: diagnosis, procedure, administrative, and hospitalization outcome variables. Each hospitalization episode is represented as a single observation containing one primary diagnosis, multiple secondary diagnoses, one primary procedure, multiple secondary procedures, finalized INA-CBG information, and length of stay (LOS). Abbreviations: ICD-10, International Classification of Diseases, Tenth Revision; ICD-9-CM, International Classification of Diseases, Ninth Revision, Clinical Modification; INA-CBG, Indonesian Case-Based Groups; LOS, Length of Stay.

Figure 3. Dataset Organization.

### 2.9 Data transformation and harmonization

Hospital administrative records are primarily generated to support clinical care and reimbursement processes, rather than for secondary research. Consequently, the extracted data require substantial transformation before they become suitable for statistical analysis, healthcare analytics, and machine learning applications. The transformation workflow was designed to preserve the original clinical information while converting heterogeneous operational records into a standardized analytical dataset. ^35^

A major challenge during repository construction was the variable number of diagnoses and procedures recorded for each hospitalization. While every patient has a principal diagnosis, the number of secondary diagnoses varies depending on the disease complexity, comorbidities, and complications. Similarly, procedural records differ considerably among patients because some admissions require only a single intervention, whereas others involve multiple diagnostic and therapeutic procedures. Such variability produces irregular data structures that are difficult to analyze using conventional statistical software or machine learning algorithms. ^36^

To address this challenge, all diagnosis and procedure records were transformed into fixed-column tabular representations. Diagnosis variables were organized into one Primary_Diagnosis column, followed by 11 secondary diagnosis variables (Secondary_diag1 to Secondary_diag11). Procedure variables were organized into one Primary_Procedure column, followed by 16 secondary procedure variables (Secondary_proc1 to Secondary_proc16). This harmonization preserved the sequential order of diagnoses and procedures while ensuring that every hospitalization episode followed an identical, structured format. Episodes containing fewer diagnoses or procedures retained empty values in the remaining columns, reflecting actual clinical documentation rather than missing or erroneous information.

The transformation process also preserved the final reimbursement information generated through the INA-CBG Grouper System. Each hospitalization episode retained its original INA-CBG code and the corresponding INA-CBG description, thereby maintaining compatibility with Indonesian case-mix reimbursement standards. Hospitalization outcome was represented using the Length of Stay (LOS) variable, calculated as the total number of inpatient days between admission and discharge. No recalculation or modification of the LOS values was performed during repository construction. ^37^

A standardized naming convention was applied to all variables to improve readability and interoperability. Diagnosis variables were prefixed consistently using Primary_ and Secondary_ identifiers, whereas procedure variables followed the same convention. This standardized structure facilitates direct import into statistical software, relational databases, Python libraries, R packages, and machine-learning frameworks without requiring additional preprocessing. Finally, all transformed variables were consolidated into a single research-ready dataset comprising 165,391 inpatient hospitalization episodes and 32 standardized variables. The resulting repository provides a harmonized representation of inpatient administrative data suitable for healthcare analytics, predictive modelling, process mining, clinical pathway analysis, and hospital operations research.

### 2.10 Repository organization

The published repository was organized to maximize transparency, reproducibility, and ease of reuse. Rather than providing only a tabular dataset, the repository includes complementary resources that enable users to understand, validate, and reproduce published results.

The repository consists of the following:

1. a de-identified CSV dataset containing all hospitalization episodes.
2. Microsoft Excel files for users who prefer spreadsheet-based analysis.
3. metadata describing each variable.
4. documentation explaining the dataset construction.
5. descriptive statistical summary.
6. visualization outputs.
7. Python scripts used during preprocessing and quality assessment; and
8. a README file describing the repository contents and recommended usage.

The principal files and supporting materials included in the published repository are listed in Table 2. This organization allows researchers with different technical backgrounds to access datasets using statistical software, programming environments, or spreadsheet applications without requiring proprietary tools.

**Table 2.** Repository contents.

File	Description
Dataset.csv	Main Dataset
Metadata.xlsx	Variable descriptions
README	Documentation
Python	Scripts

## 3. Dataset validation

Ensuring data quality is fundamental to any publicly shared research dataset. Because the present repository is intended for reuse in healthcare analytics, machine learning, and clinical pathway research, multiple validation procedures were implemented throughout the data-engineering workflow. The validation focused on structural integrity, coding accuracy, completeness, consistency, statistical plausibility, and computational reproducibility.

Unlike the validation procedures performed for predictive modelling, which typically evaluate algorithm performance, the objective of dataset validation in this study was to verify that the published repository accurately represented the original finalized administrative records while maintaining structural consistency and analytical reliability.

The validation strategy comprised six complementary components.

1. Structural validation.
2. Clinical coding validation was performed.
3. Completeness assessment of the data.
4. Consistency assessment.
5. Statistical validation; and
6. Reproducibility assessment of the proposed model.

Together, these validation procedures provide confidence that the dataset can be reliably reused for secondary analyses across multiple healthcare research areas.

### 3.1 Structural validation

Structural validation was conducted to verify that each hospitalization episode followed the predefined repository schema. Automated validation scripts were developed to examine the dataset dimensions, variable ordering, naming conventions, data types, and record uniqueness.

Each hospitalization episode was required to satisfy the following structural requirements.

1. One observation was made per hospitalization episode.
2. identical column orderings across all records.
3. standardized variable name.
4. correct the variable data types.
5. absence of duplicate observations.

The validation process confirmed that the final repository contained 165,391 unique hospitalization episodes represented by 32 standardized variables, each conforming to the predefined repository schema. Random sampling of records was performed to verify that the transformed observations remained consistent with the corresponding source records after extraction and transformation.

### 3.2 Clinical coding validation

Because the analytical value of the repository depends heavily on standardized clinical coding systems, dedicated validation procedures were applied to the diagnosis codes, procedure codes, and reimbursement classifications.

ICD-10 Diagnosis Validation

All diagnostic variables were evaluated to ensure conformity with the ICD-10 coding system. Validation included the verification of code format, removal of invalid characters, preservation of diagnosis hierarchy, and consistency between primary and secondary diagnosis fields. Records containing formatting anomalies were reviewed during preprocessing and before inclusion in the final repository.

ICD-9-CM Procedure Validation

Equivalent validation procedures were performed for the ICD-9-CM procedure codes. The validation process confirmed that the procedural codes retained their original sequence and formatting throughout the data transformation. Primary procedures were clearly distinguished from secondary procedures to preserve the procedural hierarchy.

INA-CBG Validation

The final reimbursement classifications generated using the INA-CBG Grouper System were retained without modification. Validation verified the consistency between exported reimbursement records, assigned INA-CBG codes, and corresponding textual descriptions. Only finalized reimbursement records were included, thereby minimizing inconsistencies associated with the preliminary administrative documentation.

### 3.3 Completeness assessment

Dataset completeness was evaluated to determine whether the mandatory variables required for the secondary analysis were consistently represented across hospitalization episodes.

The assessment considered the following:

1. diagnosis completeness.
2. procedure completeness.
3. INA-CBG completeness.
4. LOS completeness.

Variable-length diagnosis and procedure information naturally resulted in unused secondary diagnosis or procedure fields for less complex hospitalization events. These empty fields represent the actual absence of additional recorded diagnoses or procedures, rather than missing data. Consequently, they were intentionally preserved to maintain fidelity to original administrative records. Mandatory variables, including the principal diagnosis, finalized INA-CBG classification, textual case-mix description, and length of stay, were verified before publication to ensure complete representation of the core analytical information.

### 3.4 Consistency assessment

Beyond structural verification, an internal consistency assessment was performed to ensure that the relationships among diagnosis codes, procedure codes, case-mix classifications, and hospitalization outcomes remained unchanged throughout the repository construction process. As the dataset was intended for secondary analyses involving clinical pathway modelling, machine learning, reimbursement analysis, and healthcare operations research, preserving these relationships was considered essential.

The consistency assessment was conducted at multiple levels of the data engineering workflow. During extraction, exported hospitalization records were compared with the original Hospital Information System (HIS) to confirm that no variables were omitted or unintentionally modified. During transformation, automated scripts verified that the diagnosis variables remained associated with the corresponding procedure variables and reimbursement classifications after conversion into the standardized tabular format.

The order of the diagnosis and procedure variables was preserved throughout the transformation process. Primary diagnoses remained assigned to the Primary_Diagnosis variable, whereas additional diagnoses retained their sequential order within secondary diagnosis fields. An equivalent approach was applied to the procedural variables to ensure that primary interventions were distinguished from secondary procedures.

Consistency validation further confirmed that every hospitalization episode contained a single corresponding INA-CBG classification, together with its associated textual description and recorded length of stay. Because the repository was constructed exclusively from finalized reimbursement records, no inconsistencies were identified between reimbursement classifications and exported hospitalization episodes.

Finally, a random manual inspection of the selected hospitalization episodes was performed following automated validation. This inspection confirmed that the transformed records accurately represented the original administrative information while preserving the relationships among diagnoses, procedures, reimbursement classifications, and hospitalization outcomes.

### 3.5 Statistical validation

Statistical validation was conducted to identify potential anomalies that could indicate data corruption, extraction errors or transformation inconsistencies. Rather than evaluating predictive performance, this validation aimed to verify that the statistical characteristics of the published repository accurately reflected the original administrative data.

Descriptive statistical analyses were performed for all principal repository components, including diagnosis variables, procedure variables, INA-CBG classifications, and hospitalization outcomes.

The final repository contained:

1. 165,391 inpatient hospitalization episodes
2. 32 standardized variables
3. 606 unique INA-CBG codes
4. 823 unique INA-CBG descriptions
5. Length of stay ranging from 1 to 91 days
6. Mean LOS of 4.54 days
7. Median LOS of 4 days.

Frequency analyses were performed to examine the distribution of the diagnosis codes, procedure codes, and INA-CBG classifications. These analyses confirmed the presence of clinically plausible distributions consistent with routine inpatient hospital service. No unexpected discontinuities, abnormal spikes attributable to preprocessing errors, or duplicate hospitalization episodes were observed.

Length-of-stay values were also inspected graphically using histograms and boxplot visualizations. The resulting distributions demonstrated the expected right-skewed pattern commonly observed in inpatient administrative datasets, where most hospitalization episodes involve relatively short admissions, whereas a smaller proportion of patients experience prolonged hospitalization. This pattern provides additional confidence that the extracted hospitalization outcomes accurately represent routine clinical practice, rather than artifacts introduced during data transformation. Therefore, statistical validation confirmed that the published repository maintained the original distributional characteristics of the underlying administrative records.

Figure 4. The frequency distribution of the 20 most common INACBG classifications in the dataset. The bar lengths represent the number of hospitalization episodes assigned to each INACBG category.

**Figure 4.**
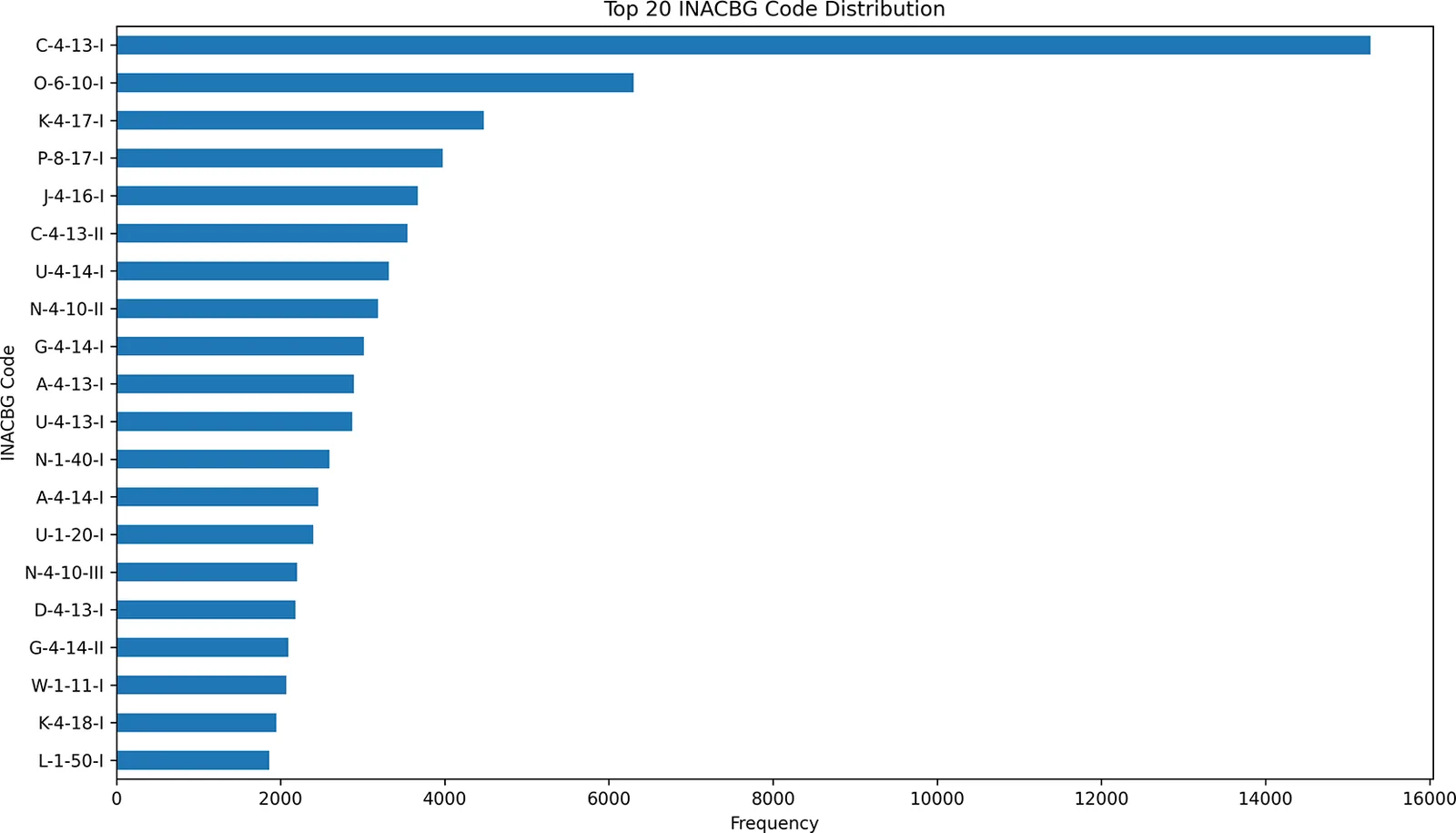
Distribution of the 20 most frequent INA-CBG classifications. The figure summarizes the most frequently assigned Indonesian Case-Based Groups within the published dataset, reflecting the diversity of inpatient reimbursement categories represented in routine hospital services. Abbreviations: INA-CBGs, Indonesian case-based groups.

Figure 4 shows the frequency distribution of the 20 most frequently occurring INACBG categories in the dataset. Each bar represents the number of hospitalization episodes associated with a specific INACBG classification. The visualization summarizes the relative frequency of case-mix categories recorded across inpatient hospitalization records and provides an overview of the distribution of hospitalization episodes within a structured dataset.

The figure includes INACBG categories representing various clinical specialties, treatment groups and severity levels. Category labels correspond to the standardized INACBG classification system, and bar lengths indicate the number of hospitalization episodes associated with each category. The frequency distribution was generated using the finalized INACBG classifications available in the source dataset and reflected the occurrence counts of hospitalization episodes assigned to each case-mix category.

Figure 5. Frequency distribution of the 20 most common INACBG descriptions in the dataset. The bar lengths indicate the number of hospitalization episodes assigned to each INA-CBG category.

**Figure 5.**
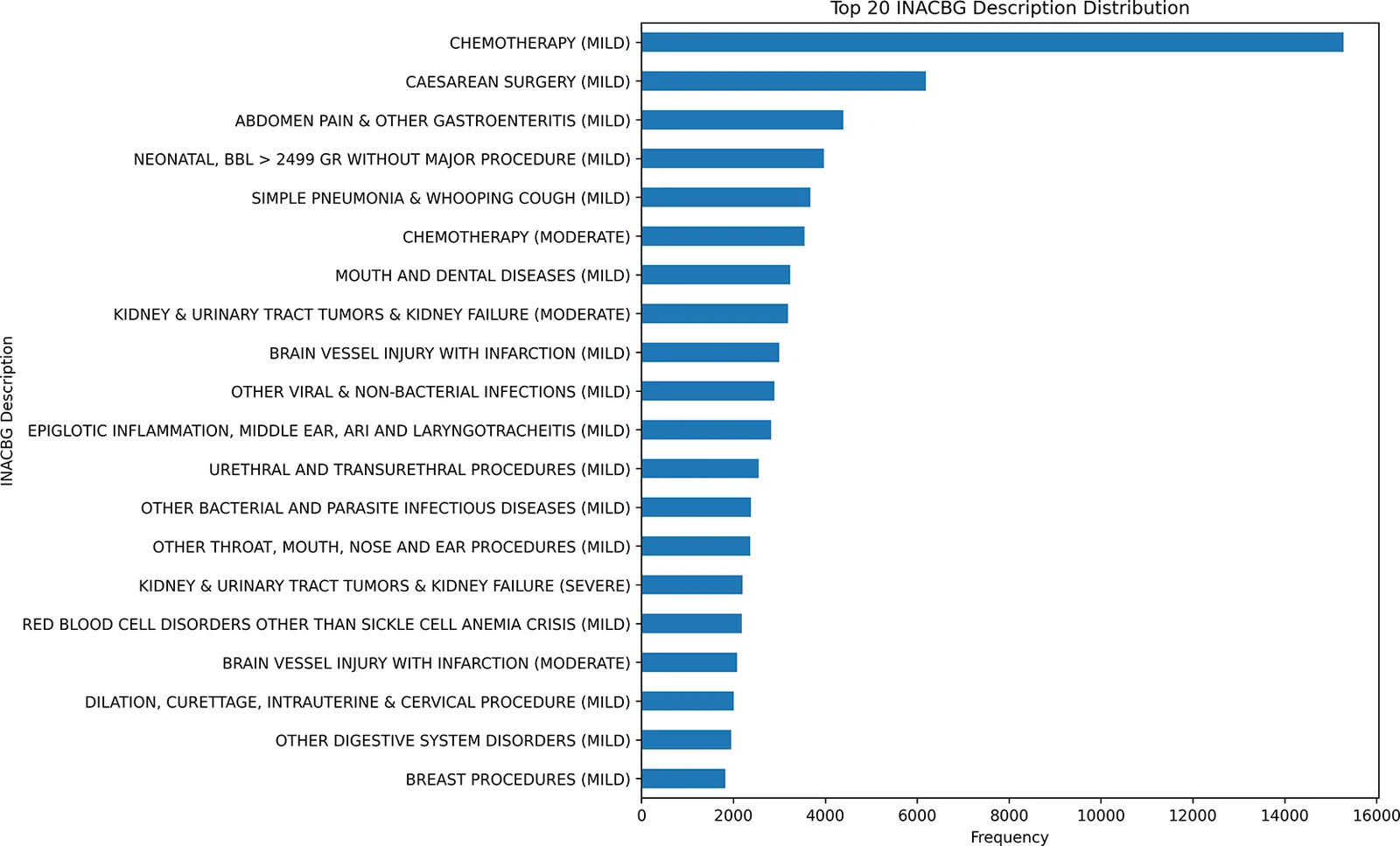
Twenty most frequent Inacbg descriptions. The figure presents the 20 most frequently occurring inacbg descriptions recorded in the published dataset, illustrating the diversity of inpatient disease categories represented within the repository. Abbreviations: INA-CBG, Indonesian Case-Based Groups.

Figure 5 shows the frequency distribution of the 20 most frequently occurring INACBG descriptions in the dataset. Each bar corresponds to a specific INACBG description and represents the number of hospitalization episodes assigned to that case-mix category. The visualization summarizes the distribution of hospitalization episodes according to their associated clinical descriptions defined within the INA-CBGs classification framework.

The displayed categories included medical, surgical, procedural, neonatal, obstetric, and specialty service classifications recorded in the dataset. Frequency counts were calculated using the finalized INACBG descriptions associated with each hospitalization episode. The figure provides an overview of the relative occurrence of the most frequently represented case-mix descriptions and documents hospitalization episodes included in the repository.

Figure 6 shows the frequency distribution of hospitalization episodes across different LOS intervals. The LOS values were measured in days and represented the duration of inpatient hospitalization episodes recorded in the dataset.

**Figure 6.**
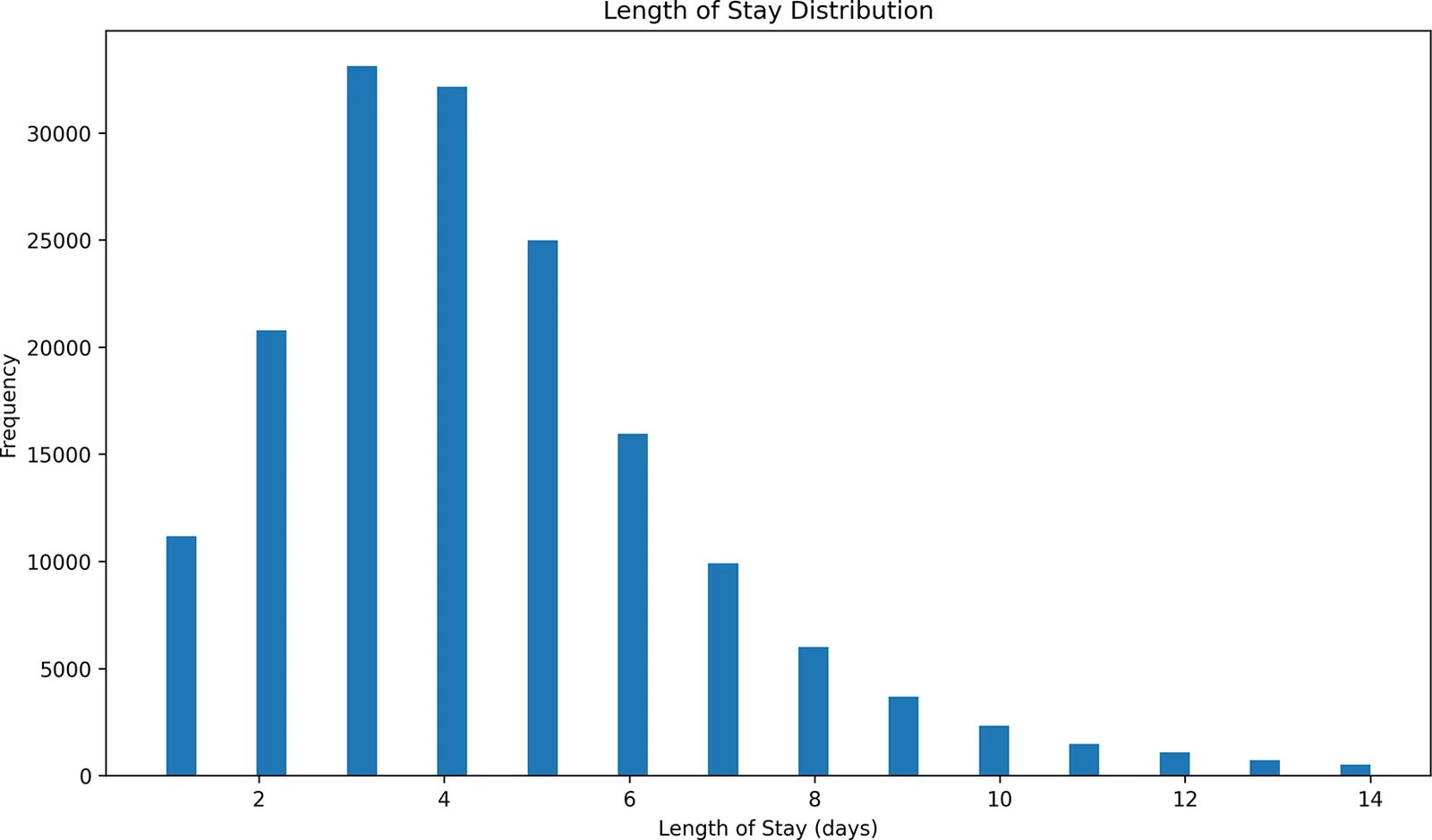
Distribution of inpatient length of stay. The histogram illustrates the frequency distribution of the hospitalization duration among all inpatient episodes in the published repository. The distribution demonstrated the expected right-skewed pattern commonly observed in hospital administrative datasets. Abbreviations: LOS, Length of Stay.

Figure 6 presents the distribution of LOS values recorded across inpatient hospitalization episodes in the dataset. LOS was measured as the number of days of hospitalization associated with each episode and was represented as a continuous numerical variable.

The histogram summarizes the frequency of hospitalization episodes across different LOS intervals and illustrates the overall distribution of hospitalization durations. Each bar represents the number of episodes within a specific LOS. The visualization was generated using LOS values extracted from the finalized hospitalization records and provided a descriptive overview of the hospitalization duration information available in the dataset. The LOS variable was included as a dedicated outcome variable and recorded for all hospitalization episodes. A histogram is provided to document the range and distribution of hospitalization durations represented within the dataset and to familiarize users with the structure of the LOS data available in the repository.

Figure 7. Length-of-stay distribution across the top 10 INACBG categories. Boxplots show the distribution of LOS values for the ten most frequently occurring INACBG classifications in the dataset. Each boxplot summarizes the median, interquartile range (IQR), minimum values, and maximum values recorded for hospitalization episodes within the corresponding INACBG categories.

**Figure 7.**
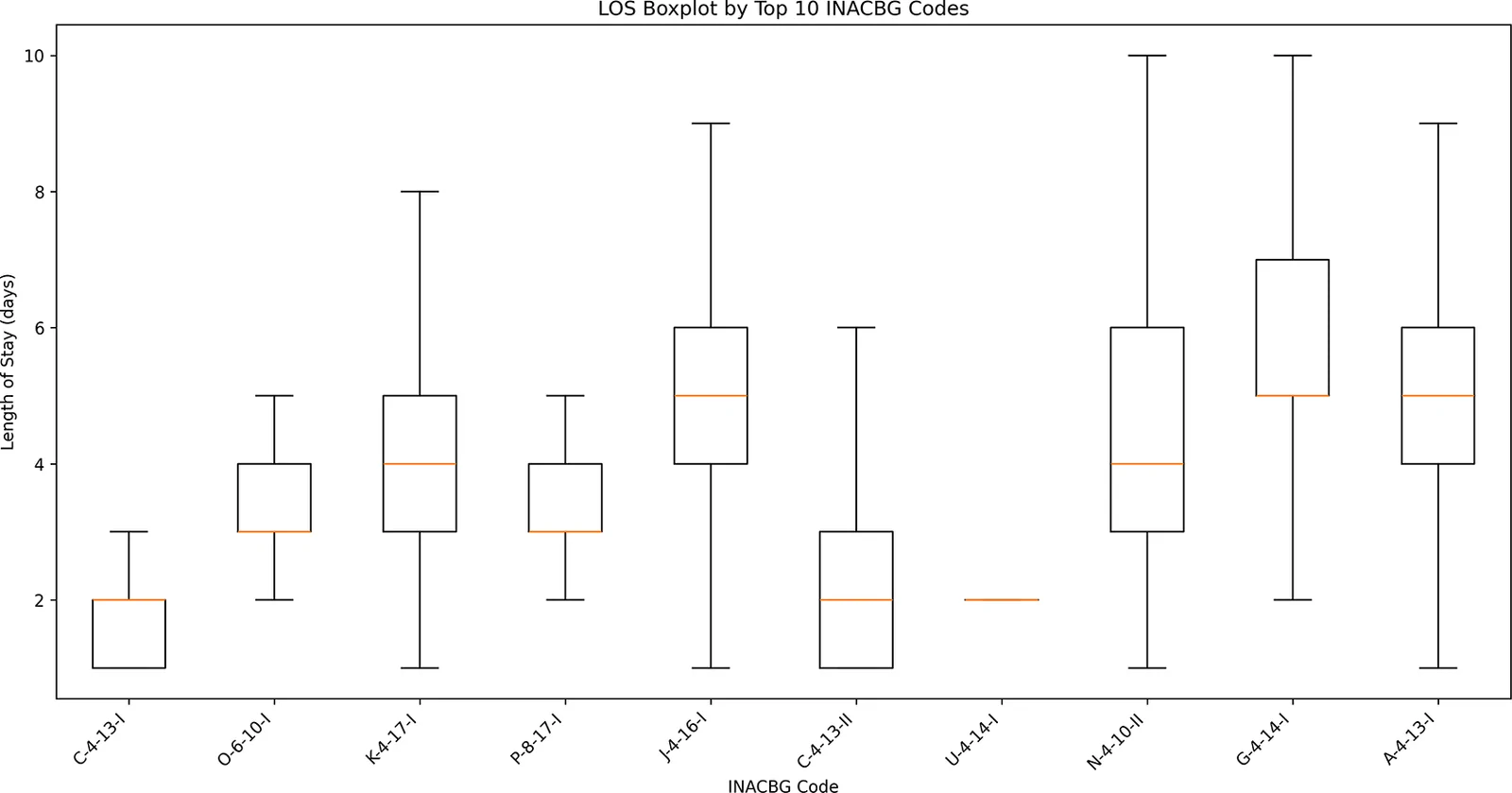
Length-of-stay distribution across the top 10 INACBG categories. Boxplots show the distribution of LOS values for the ten most frequently occurring INACBG classifications in the dataset. Each boxplot summarizes the median, interquartile range (IQR), minimum values, and maximum values recorded for hospitalization episodes within the corresponding INACBG category. Abbreviations: INA-CBG, Indonesian Case-Based Groups; LOS, length of stay.

Figure 7 presents boxplots summarizing the distribution of LOS values across the ten most frequently occurring INACBG categories in the dataset. Each boxplot corresponds to a specific INACBG classification and displays the distribution of hospitalization duration associated with that category. The boxplots provide a graphical representation of the LOS values by showing the median, IQR, minimum, and maximum values recorded within each INACBG category. The visualization was generated using hospitalization episodes assigned to the most prevalent case-mix classifications identified in the dataset.

The LOS values are represented in days and were extracted from the final hospitalization records. The figure documents the range and distribution of hospitalization durations associated with frequently occurring INACBG categories and provides an overview of the variability in LOS across case-mix classifications represented within the repository. This visualization describes the structure of the LOS variable within the major INACBG categories and familiarizes users with the available data on hospitalization duration in the dataset.

Figure 8. The heatmap shows the frequency of co-occurrence between the diagnosis and procedure categories in the dataset. Rows and columns represent the diagnosis and procedure categories, respectively, and the color intensity corresponds to the relative frequency of diagnosis–procedure combinations observed across hospitalization episodes.

**Figure 8.**
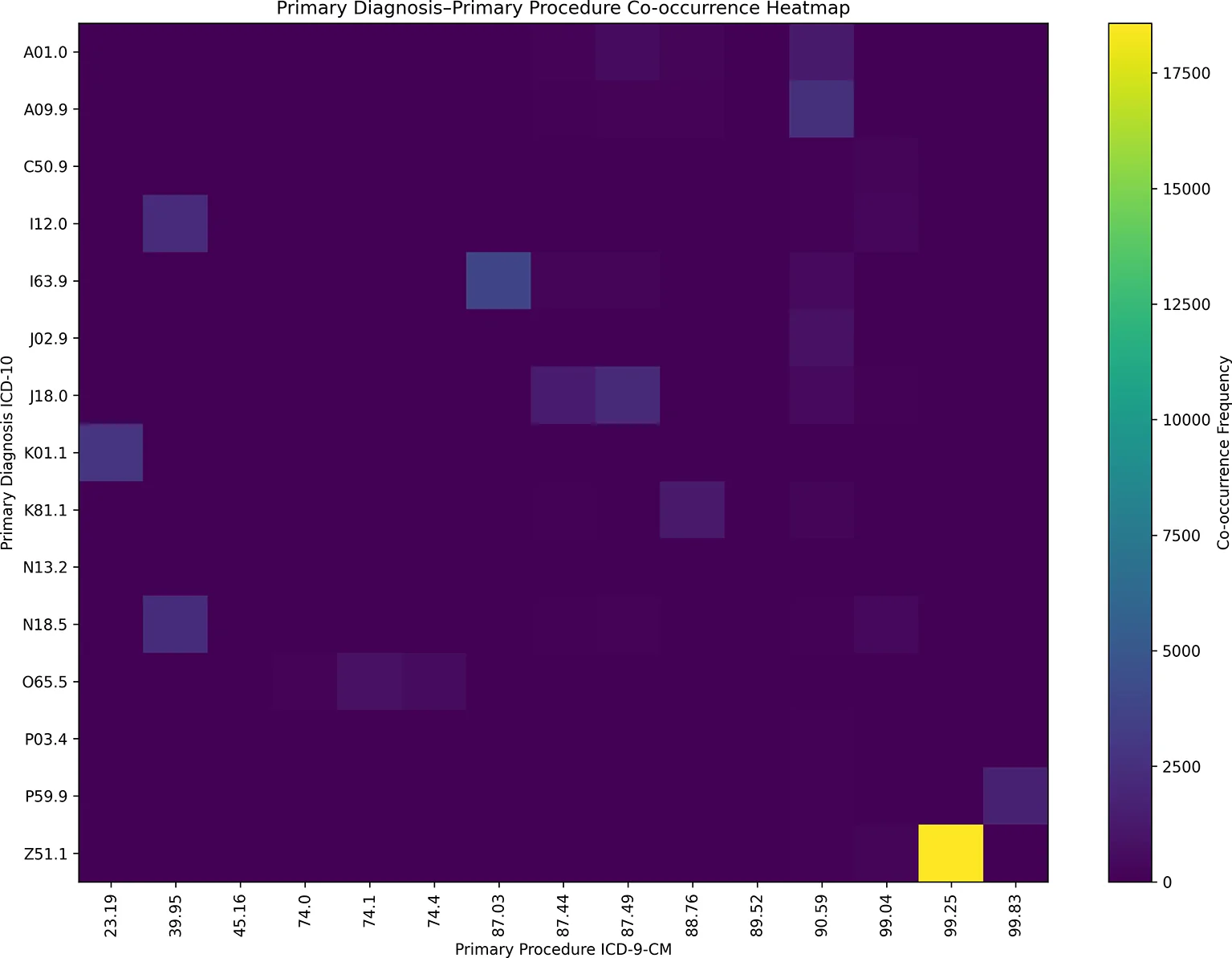
Heatmap of diagnosis–procedure co-occurrence. The heatmap visualizes the frequency of co-occurrence between diagnosis and procedure codes recorded during inpatient hospitalization episodes, providing an overview of the clinical coding relationships contained within the repository. Abbreviations: ICD-10, International Classification of Diseases, Tenth Revision; ICD-9-CM, International Classification of Diseases, Ninth Revision, Clinical Modification.

Figure 8 presents a heatmap summarizing the frequencies of co-occurrence between the diagnosis and procedure codes recorded in the dataset. The visualization was generated using selected diagnosis and procedure categories identified from inpatient hospitalization episodes and displayed the frequency of specific diagnosis–procedure combinations within the repository.

The rows and columns represent the diagnosis and procedure categories, respectively. The color intensity corresponds to the relative frequency of co-occurrence between the diagnosis and procedure categories, with darker and lighter color gradients indicating different frequencies of co-occurrence. The heatmap was constructed from the diagnosis and procedure variables coded using the ICD-10 and ICD-9-CM classification systems.

The visualization provides a structured representation of the diagnosis–procedure combinations in the dataset and documents the available occurrence patterns within the hospitalization records. This figure is included to familiarize users with the relationships between the diagnosis and procedure variables in the repository and to illustrate the structure of the diagnosis–procedure associations in the dataset. The overall characteristics of the final dataset are summarized in Table 3.

**Table 3.** Dataset characteristics.

Characteristic	Value
Inpatient Hospitalization Episodes	165,391
Total Variables	32
Unique Primary ICD-10 Diagnosis Codes	3,247
Unique ICD-10 Diagnosis Codes (All Diagnosis Variables)	8,912
Unique Primary ICD-9-CM Procedure Codes	1,186
Unique ICD-9-CM Procedure Codes (All Procedure Variables)	3,754
Unique INA-CBG Codes	606
Unique INA-CBG Descriptions	823
Mean Length of Stay (days)	4.54
Median Length of Stay (days)	4
Standard Deviation of Length of Stay	2.82
Minimum Length of Stay (days)	1
25th Percentile (Q1)	3
75th Percentile (Q3)	6
95th Percentile	9
99th Percentile	14
Maximum Length of Stay (days)	91

### 3.6 Reproducibility assessment

Reproducibility is a fundamental requirement for reusable scientific data sets. Accordingly, the repository was designed to ensure that independent researchers can reproduce every stage of the published data engineering workflow using openly available resources.

The public repository contains not only the processed dataset but also the supplementary materials required to reproduce the repository construction, including Python scripts, preprocessing workflows, metadata, documentation, descriptive statistical summaries, and visualization outputs. The computational workflow relies exclusively on open-source software libraries, thereby avoiding dependence on proprietary analytical platforms.

To facilitate independent verification, variable names were standardized using consistent naming conventions, documentation was provided for each dataset component, and the software environment used during the repository construction was explicitly reported. The inclusion of metadata describing each variable further improved interoperability with statistical software, database systems, and machine learning frameworks.

By combining openly accessible data, reproducible computational workflows, and comprehensive documentation, the repository satisfies the essential principles of transparency and enables reuse in healthcare analytics, artificial intelligence, clinical pathway analysis, hospital operations research, and methodological benchmarking of these fields.

### 3.7 Summary of dataset validation

To provide a comprehensive overview of the quality assurance process, the validation procedures performed during dataset construction are presented in Table 4. Validation was conducted at multiple stages of the data engineering workflow to ensure that the published repository accurately represented the original inpatient administrative records while maintaining structural integrity, coding consistency, completeness, and reproducibility. The validation framework encompassed structural verification, clinical coding assessment, completeness evaluation, consistency checking, statistical validation, computational reproducibility and repository verification.

**Table 4.** Summary of data quality and validation procedures.

Validation Category	Validation Procedure	Result	Purpose
Structural Validation	Verification of dataset dimensions	165,391 records; 32 variables	Ensure structural integrity
Variable Naming	Standardized variable names	Passed	Improve interoperability
Duplicate Records	Duplicate hospitalization episodes	None detected	Ensure record uniqueness
ICD-10 Validation	Diagnosis code format verification	Passed	Preserve diagnosis consistency
ICD-9-CM Validation	Procedure code verification	Passed	Preserve procedure consistency
INA-CBG Validation	Consistency between code and description	Passed	Maintain reimbursement integrity
LOS Validation	Verification of hospitalization duration	Complete for all records	Preserve outcome variable
Missing Value Assessment	Mandatory variable completeness	Complete for mandatory variables	Dataset completeness
Data Type Validation	String/integer format verification	Passed	Prevent import errors
Statistical Validation	Frequency distribution assessment	No abnormal distributions observed	Detect transformation anomalies
Reproducibility	Workflow reproducibility using Python scripts	Passed	Support open science
Repository Validation	Verification of Zenodo repository contents	Dataset, documentation, metadata, and scripts available	Enable data reuse

Structural validation confirmed that the final repository consisted of 165,391 unique inpatient hospitalization episodes organized into 32 standardized variables, with no duplicate records or inconsistencies in variable organization. Clinical coding validation verified that the diagnosis and procedure variables complied with the ICD-10 and ICD-9-CM coding standards, while administrative validation confirmed the consistency between the INA-CBG codes and their corresponding descriptions. In addition, the completeness assessment demonstrated that all mandatory variables required for secondary analyses, including primary diagnosis, INA-CBG classification, and length of stay, were available throughout the dataset.

Statistical validation further confirmed that the published repository preserved the original characteristics of administrative records without introducing unexpected anomalies during data transformation. Finally, the inclusion of metadata, documentation, and reproducible Python scripts ensures that the complete workflow can be independently verified and reused by other researchers in the field. Collectively, these validation procedures demonstrate that the dataset satisfies the essential requirements of a high-quality, reusable healthcare research repository suitable for healthcare analytics, clinical pathway analysis, machine learning, process mining, reimbursement research, and hospital operations management.

### 3.8 Strengths and limitations of the dataset

The published repository possesses several characteristics that distinguish it from existing healthcare administrative datasets.

First, the dataset integrated three complementary standardized clinical coding systems within a single analytical framework. The combination of ICD-10 diagnoses, ICD-9-CM procedures, and finalized INA-CBG reimbursement classifications enables comprehensive investigations into diagnosis–procedure relationships, case-mix characteristics, and hospitalization outcomes without requiring additional linkage between databases.

Second, the repository comprises 165,391 inpatient hospitalization episodes, representing approximately ten years of routine inpatient services. The large sample size improves the statistical power and supports applications involving descriptive epidemiology, clinical pathway analysis, machine learning, process mining, explainable artificial intelligence, reimbursement research, and hospital resource planning.

Third, all hospitalization episodes originated from finalized reimbursement records rather than preliminary clinical documentation. Consequently, diagnosis codes, procedure codes, case-mix classifications, and hospitalization outcomes represent validated administrative information routinely used in the national health insurance reimbursement process.

Fourth, the repository has been transformed into a harmonized fixed-column tabular structure that can be directly imported into common analytical software packages without complex preprocessing. This substantially lowers the technical barrier for researchers interested in secondary analyses of hospital administrative data.

Despite these strengths, several limitations should be acknowledged in this study. The repository was derived from a single tertiary referral hospital and therefore reflects local clinical practice, coding behavior, and reimbursement policies. Consequently, external validation may be required before extrapolating the findings to different healthcare systems or countries.

Furthermore, patient demographic identifiers, laboratory results, medication records, physiological measurements, radiological findings, and free-text clinical narratives were intentionally excluded to preserve patient privacy and to comply with institutional data-sharing policies. Therefore, the repository is primarily intended for studies involving healthcare administration, reimbursement analysis, clinical pathway modelling, and hospital operations rather than investigations requiring detailed clinical or physiological information.

Finally, because the dataset represents routinely collected administrative information, its quality depends on the accuracy of clinical documentation and coding practices performed during routine healthcare delivery. Nevertheless, restricting repository construction to finalized reimbursement records substantially reduces the likelihood of incomplete or inconsistent administrative data.

## 4. Ethical considerations

This retrospective study used administrative hospitalization data collected during routine clinical practices. Before repository construction, all direct patient identifiers were permanently removed using a structured de-identification process. The resulting analytical repository contained no personally identifiable information and was used exclusively for scientific research purposes.

This study was conducted in accordance with the principles of the Declaration of Helsinki. The research protocol was reviewed and approved by the Health Research Ethics Committee (HREC), Universitas Muhammadiyah PKU (UMPKU) Surakarta, under Ethical Clearance No. 231/LPPM/UMPKU/VI/2026 (Protocol ID: 26/06/228), issued on June 8, 2026.

Because this study involved a retrospective analysis of fully de-identified administrative healthcare records, no direct patient contact occurred, and the requirement for individual informed consent was waived in accordance with institutional ethical regulations governing the secondary use of anonymized healthcare data. The publicly released repository contains only anonymized variables and complies with institutional requirements for ethical data sharing and scientific reuse of data.

## 5. FAIR Data Principles

The repository was developed following the principles of Findability, Accessibility, Interoperability, and Reusability (FAIR) to maximize its value for the scientific community. These principles improve the discoverability and long-term usability of research datasets and have become widely accepted standards for scientific data publications.

Findability

The dataset was deposited in a publicly accessible Zenodo repository and assigned a persistent Digital Object Identifier (DOI), enabling long-term citation and retrieval. Metadata describing the dataset, repository contents, variable definitions, and documentation are provided to facilitate discovery by researchers and indexing services.

Accessibility

All repository contents are openly available without subscription or institutional access restrictions. Users can directly download the dataset, documentation, and accompanying source code from the repository using a permanent DOI. No additional permissions are required for academic re-use.

Interoperability

The repository employs internationally recognized healthcare coding systems, including ICD-10 and ICD-9-CM, along with the Indonesian Case-Based Groups (INA-CBG) reimbursement classification. The dataset variables are organized in standard CSV and Microsoft Excel formats that can be imported into most statistical software, database systems, and machine learning environments.

Reusability

The repository includes metadata, documentation, pre-processing scripts, descriptive statistics, visualization outputs, and detailed methodological descriptions of every stage of repository construction. These supplementary materials enable independent researchers to reproduce the published workflow and reuse the dataset in future studies involving healthcare analytics, artificial intelligence, clinical pathway analysis, process mining, reimbursement research and hospital operations.

## Data Availability

The complete dataset and supporting materials are publicly available through the Zenodo research data repository. Repository title: Clinical Pathway Patterns Based on Case Mix Groups [Dataset]. Repository: Zenodo. Persistent DOI: 10.5281/zenodo.22135469. ^30^ The repository contains the de-identified inpatient hospitalization dataset, metadata and variable descriptions, Python preprocessing and validation scripts, statistical summaries, documentation describing repository construction, and README documentation. Zenodo: Clinical Pathway Patterns Based on Case Mix Groups [Dataset]. https://doi.org/10.5281/zenodo.22135469. ^30^ The same Zenodo repository contains the supporting materials associated with this Data Note, including metadata and variable descriptions, Python scripts used for preprocessing and data validation, statistical summaries, repository documentation, and README documentation https://doi.org/10.5281/zenodo.22135469. ^30^ Data are available under the terms of the Creative Commons Attribution 4.0 International license (CC-BY 4.0).
