# Supplementary material for: A de-identified inpatient clinical pathway dataset integrating ICD-10 diagnoses, ICD-9-CM procedures, Indonesian Case-Based Groups (INA-CBG), and length of stay for healthcare analytics
Source: F1000Res. 2026 Oct 14;15:1713. [Version 1] doi: 10.12688/f1000research.187759.1 (PMC13731902; doi:10.12688/f1000research.187759.1)
Supplement: Peer review history

# Reviewer report: Reviewer response for version 1

**DOI:** 10.5256/f1000research.207226.r532723

**Published:** 2026-11-03

**Authors:** Hariadi Yutanto

**Affiliations:** 1 Universitas Hayam Wuruk Perbanas, Surabaya, East Java, Indonesia

Summary

 This data note describes a de-identified inpatient administrative dataset from an Indonesian tertiary hospital (November 2016–April 2026). It contains 165,391 episodes and 32 variables combining ICD-10 diagnoses, ICD-9-CM procedures, INA-CBG classifications, and length of stay (LOS). Publicly available data linked to the Indonesian case-mix system are scarce, and this dataset is a welcome contribution, particularly for health services research in LMIC settings.

 I downloaded the Zenodo deposit, ran the authors' script (preprocessing_validation.py), and performed some additional checks on dataset_cp_fix_2.xlsx. The authors' script reproduced exactly the figures in their validation_report.csv and statistical_summary.csv. The dimensions (165,391 × 32), the INA-CBG code and description counts (606 and 823), and all LOS statistics in Table 3 (mean 4.54, median 4, SD 2.82, range 1–91 days) were also reproduced exactly. Mandatory fields (primary diagnosis, INA-CBG code, description, and LOS) are complete for all records. All ICD-10 codes are consistently formatted, and secondary diagnosis and procedure fields are filled contiguously, without gaps in sequence. The authors are to be commended for this.

 I have a few minor suggestions that would improve the alignment between the manuscript and the deposited data and make the dataset easier to reuse correctly. None of them requires new data collection.

 Minor suggestions for the authors

 1. Duplicate records. Table 4 states that no duplicate records were detected, whereas the deposited data and the authors' own validation_report.csv show 52,286 exact duplicate rows. These most likely represent legitimate repeated admissions with identical coded content. For example, 16,148 of the 19,156 episodes with Z51.1 as the primary diagnosis are exact duplicates, and the pattern Z51.1 / C50.9 / 99.25 / C-4-13-I / LOS 1 appears 3,874 times, consistent with chemotherapy cycles. A brief correction of Table 4 and Section 3.1, explaining that identical rows can represent distinct admissions, would be helpful.

 2. Format of procedure codes. The ICD-9-CM procedure columns are stored as numeric cells in the XLSX file. As a result, 3,501 entries appear without their leading zero (e.g., 0.09, 2.34, 6.2 instead of 00.09, 02.34, 06.2), and codes ending in zero may lose their final digit (e.g., 96.60 shown as 96.6). Saving these columns as text, and providing a CSV version, is recommended so that users obtain the correct codes.

 3. Code counts in Table 3. My counts from the deposited data differ from those reported: unique primary ICD-10 codes 2,931 (Table 3: 3,247); unique ICD-10 codes across all diagnosis fields 4,240 (Table 3: 8,912); unique primary ICD-9-CM codes 1,076 (Table 3: 1,186); unique ICD-9-CM codes across all procedure fields 1,414 (Table 3: 3,754). Please verify and update these figures.

 4. INA-CBG descriptions. 215 of the 606 INA-CBG codes appear with more than one description, mostly spelling or translation variants (e.g., "SEPTICEMIA"/"SEPTYKEMIA" and "COMPLEX BILDICULOSIS PROCEDURE"/"COMPLEX BILE DUCT PROCEDURE"). One pair has opposite meanings (B-1-12-I: "NON-LAPAROSCOPY CHOLECYSTECTOMY" vs "LAPAROSCOPIC NON-CHOLESISTECTOMY"). In addition, 15 descriptions are associated with more than one code (e.g., "CAESAREAN SURGERY (MILD)" with O-6-10-I and O-6-13-I), and in 12 records the severity level of the code (I/II/III) does not match the severity label in the description (e.g., L-4-12-III with "(MILD)"). This explains the 823 descriptions for 606 codes. Harmonizing descriptions to the official INA-CBG nomenclature, or adding a code–description mapping table and adjusting the corresponding line in Table 4 would be helpful.

 5. Length of stay beyond 14 days. The LOS histogram (Figure 6) displays values up to 14 days, while 1,550 episodes have a longer stay (87 exceed 30 days). Noting this in the caption would give readers a complete picture of the distribution.

 6. De-identification. In the deposited data, 104,728 of 165,391 rows (about 63%) are unique combinations of the published variables. A short statement on how disclosure risk was considered, for instance, for rare diagnosis–procedure combinations or very long stays, would strengthen the de-identification section.

 Conclusion

 This is a useful and well-prepared dataset that fits the scope of a data note. Its structure, completeness, and LOS statistics were reproduced exactly from the deposit, and I consider it an acceptable contribution. I recommend that the authors address the minor points above in a revised version, in particular saving procedure codes as text and aligning Tables 3 and 4 with the deposited data, so that users can reuse the dataset correctly.

 Use of AI: Claude Opus 5.5 (Anthropic) was used to support this review in three ways: to identify potential issues in the published article and the publicly available Zenodo files, to write a Python script for checking the deposited dataset, and to assist with English wording. I ran the authors' script and the additional checks myself, and all quantitative findings in this report come from that output. Points that my own checks did not support were removed. The judgements, approval status, and final content of this report are my own. The tool was used to support a thorough consistency check between the article and the dataset and to improve clarity of English.

Are sufficient details of methods and materials provided to allow replication by others?

Yes

Is the rationale for creating the dataset(s) clearly described?

Yes

Are the datasets clearly presented in a useable and accessible format?

Partly

Are the protocols appropriate and is the work technically sound?

Yes

Reviewer Expertise:

Health information systems, data analytics, and information technology. I assessed the structure, reproducibility, and internal consistency of the deposited dataset against the manuscript; I did not assess the clinical accuracy of the coding against source medical records.

I confirm that I have read this submission and believe that I have an appropriate level of expertise to confirm that it is of an acceptable scientific standard.

# Reviewer report: Reviewer response for version 1

**DOI:** 10.5256/f1000research.207226.r532721

**Published:** 2026-10-23

**Authors:** Siti Maesaroh

**Affiliations:** 1 Universitas Mayasari Bakti, Tasimalaya, West Java, Indonesia

This Data Note describes a substantial inpatient administrative dataset integrating ICD-10 diagnoses, ICD-9-CM procedures, Indonesian Case-Based Groups (INA-CBG), and length of stay. The dataset comprises 165,391 hospitalization episodes and 32 variables, covering records collected between November 2016 and April 2026. Its principal contribution is the availability of an Indonesian administrative healthcare resource that can support secondary analyses of diagnosis–procedure associations, case-mix patterns, and hospital utilization.

 The manuscript is appropriate for the Data Note article type. Its contribution lies in dataset construction, documentation, and reuse rather than hypothesis testing or predictive model development. The objectives are clearly stated, and the methodological description provides a useful account of the administrative context underlying the records.

 Strengths

 The manuscript explains the source workflow, eligibility criteria, extraction procedures, de-identification process, and transformation into a consistent tabular structure. Preserving primary and secondary diagnosis and procedure fields supports analyses at different levels of coding detail.

 A particularly useful distinction is made between unused secondary fields and missing mandatory information. This clarification helps prevent inappropriate imputation and supports more faithful interpretation of administrative records.

 The validation framework addresses structural integrity, coding formats, completeness, internal consistency, statistical plausibility, and reproducibility. Table 3 provides informative descriptive characteristics, while Table 4 consolidates the reported quality assurance procedures.

 Ethical approval and the consent waiver are explicitly documented. The manuscript also acknowledges important limitations, including its single-institution origin, dependence on routine coding practices, and absence of detailed demographic and physiological information. These limitations appropriately define the scope of potential reuse.

 Minor comments for clarification

 Scope of pathway analysis. The diagnosis and procedure fields preserve administrative coding order, which should be distinguished from the chronological sequence of clinical events. Without event timestamps, the dataset primarily supports episode-level pathway patterns and co-occurrence analyses; conventional temporal process mining would require additional information.

 Episode uniqueness. A brief explanation of how duplicate hospitalization episodes were identified before identifiers were removed would strengthen the documentation. Different admissions can legitimately share the same combination of published variables.

 INA-CBG descriptions. The reported 606 unique INA-CBG codes and 823 descriptions would benefit from a short explanation of whether the difference reflects terminology updates, textual variation, or other administrative factors.

 Presentation and terminology. Section 2.1 lists the fourth workflow stage as “(6),” which should be corrected to “(4).” LOS should also be described consistently as an integer-valued duration recorded in days. Statements about reproducibility should distinguish analyses reproducible from the released dataset from extraction steps requiring access to the protected source system.

 These comments are intended to improve precision and guide responsible reuse. They do not alter the overall positive assessment of the dataset’s contribution.

 Final decision: Approved

 The manuscript presents a useful and clearly described administrative healthcare dataset with a documented construction workflow, reported quality checks, and explicit ethical oversight. Within its stated scope and limitations, it provides a worthwhile resource for healthcare analytics and methodological research.

Are sufficient details of methods and materials provided to allow replication by others?

Yes

Is the rationale for creating the dataset(s) clearly described?

Yes

Are the datasets clearly presented in a useable and accessible format?

Yes

Are the protocols appropriate and is the work technically sound?

Yes

Reviewer Expertise:

Information Technology, Artificial Intelligence, Smart System, Smart City, Information System

I confirm that I have read this submission and believe that I have an appropriate level of expertise to confirm that it is of an acceptable scientific standard.
